# Gender Differences in Dendritic Damage, Gut Microbiota Dysbiosis, and Cognitive Impairment During Aging Processes

**DOI:** 10.1111/cns.70164

**Published:** 2024-12-26

**Authors:** Yuan‐Yuan Han, Kang Li, Jing‐Yu Hu, Ji‐Chao Wu, Xiao Li, De‐Xiang Liu, Chu‐Hua Li

**Affiliations:** ^1^ Department of Radiology The Affiliated Panyu Central Hospital of Guangzhou Medical University Guangzhou China; ^2^ School of Life Science South China Normal University Guangzhou China; ^3^ Department of Neurology, Sun Yat‐sen Memorial Hospital Sun Yat‐sen University Guangzhou China

**Keywords:** aging, cognitive impairment, dendritic damage, gender differences, gut microbiota dysbiosis

## Abstract

**Background:**

Cognitive impairment is a common and feared characteristic of aging processes, and one key mechanism of cognition is hippocampal synaptic structure. Previous studies have reported that gut microbiota dysbiosis occurred in neurodegenerative diseases and other brain disorders with cognitive impairment. However, it is not clear how gender differences affect cognitive impairment in aging processes and whether they affect synaptic structure and gut microbiota. Here, we studied the gender differences in cognitive ability, dendritic morphology, and gut microbiota of adult, middle‐, and old‐aged rats.

**Methods:**

The cognitive ability of rats using was assessed by the Y‐maze SAB test, the light/dark discrimination test, and the MWM test. Dendritic morphology was investegated by Golgi staining. Microbiota composition, diversity and richness were analyzed by 16S rRNA gene sequencing.

**Results:**

The results showed that the cognitive ability of old‐aged rats was decreased than adult and middle‐aged rats in the spontaneous alternation behavior test, the light/dark discrimination test in Y‐maze, and the MWM test; males have better cognitive ability than the females for middle‐aged rats. The neuronal dendritic structures of CA1, CA3, and DG regions of the hippocampus were damaged to different degrees during aging, and the spine loss of females was more than that of males in CA1 and CA3 of middle‐aged rats. In addition, the microbial diversity of gut microbiota was significantly decreased in old‐aged male rats; the distribution and composition of microbiota communities were different between male and female rats at different ages.

**Conclusion:**

These findings revealed that cognitive impairment in aged rats might result from dendritic damage in the hippocampus and gut microbiota dysbiosis, which provides direct evidence that gender differences in dendritic damage and gut microbiota dysbiosis might associate with cognitive impairment in naturally aged rats.

## Graphical abstract

The present study showed the cognitive ability of old‐aged rats was decreased than adult and middle‐aged rats; males have better cognitive ability than females in middle‐aged rats. The neuronal dendritic structures of the hippocampus were damaged to different degrees during aging, and spine loss occurred in females rather than males in middle‐aged rats. In addition, the microbial diversity of gut microbiota was significantly decreased in old‐aged male rats; some specific bacteria of microbiota communities were changed with aging.

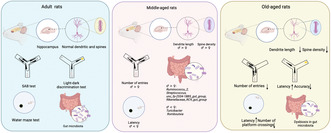

## 1 Introduction

Aging is characterized by a progressive decline in function, including organ function decline [1] and cognitive impairment [2]. Cognitive impairment is a common and feared characteristic of aging processes. Age‐related cognitive impairment is highly complex, and the mechanisms remain largely unclear. Previous studies have shown that the mechanisms of age‐related cognitive impairment include abnormal regeneration of stem cells [3] and changes in hippocampal neuronal morphology [4]. The hippocampus strongly correlates with cognitive impairment, especially for spatial memory. It is made up of three regions: the cornu ammonis (CA1, CA2, and CA3), the dentate gyrus (DG), and the subiculum. Previous studies have revealed that a reduction in dendrites branch and loss of spines has been demonstrated in the hippocampus of rodents with cognitive impairment [5, 6].

Many reports have proved that gut microbiota plays a crucial role in the occurrence and development of many brain diseases [7]. The gut microbiota affects cognition by regulating brain‐derived neurotrophic factor (BDNF) expression, the quantity of dopamine and activation of serotonin synthesis pathways, tryptophan metabolism, neuropeptide Y system, oxytocin, and vasopressin [8]. Due to the increasing evidence of a bidirectional communication system between the central nervous system and the gastrointestinal tract, it is often referred to as the “gut–brain axis.” As we age, the composition and function of gut microbiota changes in humans [9]. Although there is an established relationship between the gut dysbiosis and brain senescence in the scientific literature, the data are conflicting and varied in many cases. Recent studies showed that fecal microbiota transplantation from older rodent donors resulted in impaired cognition and synaptic structure in young adult recipients [10, 11]. Conversely, transplantation of gut microbiota from healthy elderly donor mice into young sterile recipient mice enhanced hippocampal neurogenesis [12]. A clinical study showed that aging in healthy donors is associated with compositional alterations in the fecal microbiome without change in the overall microbial diversity [13]. Besides, there are no alternative microbiota‐related drugs to modulate gut dysbiosis to promote neurodegenerative cognitive function in humans. Therefore, it is an urgent need for deeper and more extensive research on gut dysbiosis and cognitive impairment caused by aging.

Both human and experimental animal research have revealed considerable gender differences in the composition of gut microbiota [14, 15, 16]. Interestingly, there was a marked difference in the decline of cognitive function between genders. Women are twice as likely to have Alzheimer's disease as men [17]. Microglia in female mice are more easily activated to produce some genes that are strongly enriched in Alzheimer's disease [18], which suggested that gender was a risk factor for cognitive impairment in the brain. It is unclear whether gender differences in gut microbiota contribute to gender differences in aging‐related cognitive impairment. Therefore, we studied the gender differences in cognitive ability, dendritic morphology, and gut microbiota of adult, middle‐, and old‐aged rats, which provide direct evidence for the physiological mechanism of gender differences in cognitive impairment caused by aging.

## 2 Materials and Methods

### 2.1 Animals

**FIGURE 1:**
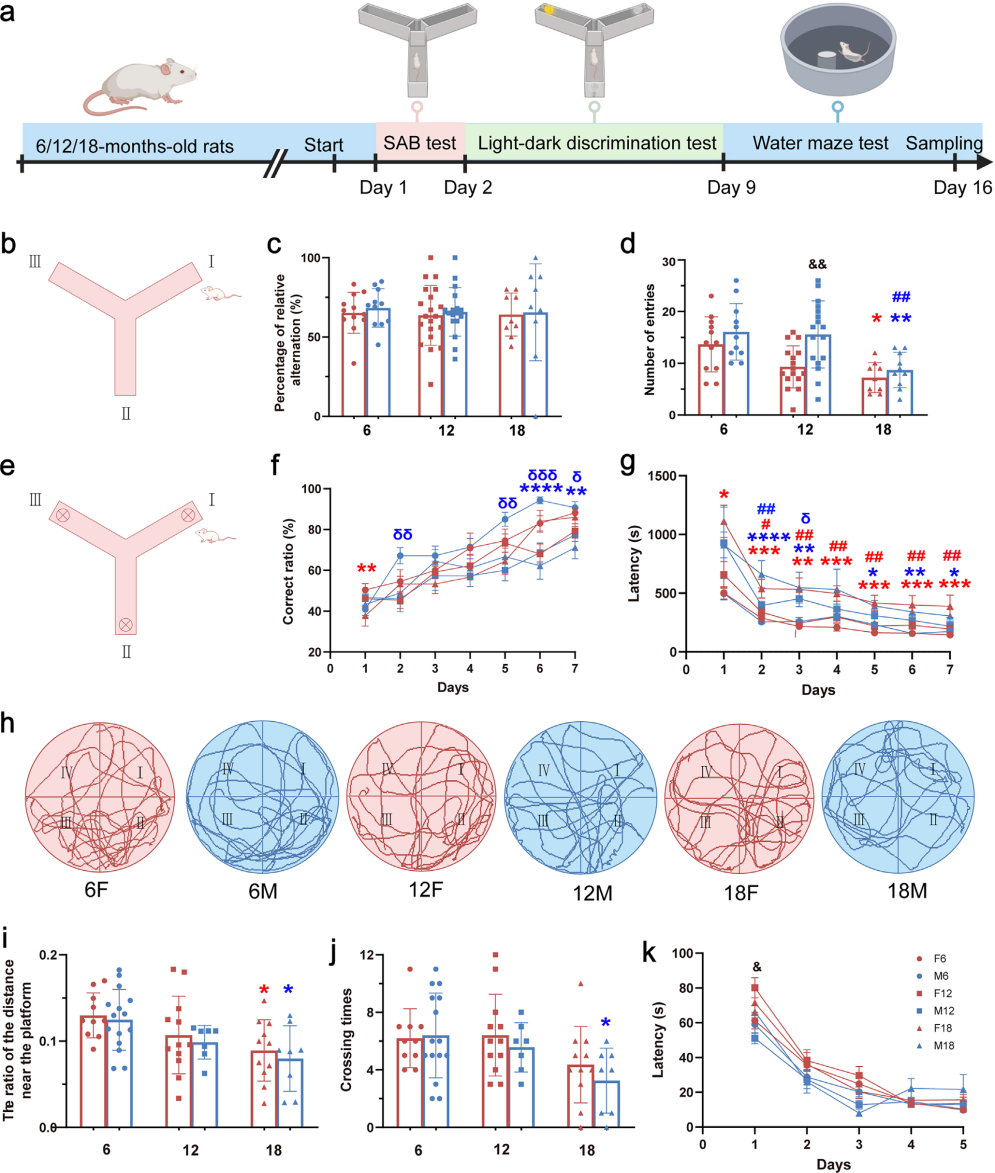
Effects of age and sex on behavioral tests in rats. (a) Timeline of behavior test. (b) SAB test schematic diagram. (c) The percent of relative alternation in rats. (a), (d) The total number of entries in rats. (e) Light–dark discrimination schematic diagram. (f) Correct rate of rats. (g) Latency of rats. (h) Water maze traces. (i) The ratio of the distance near the platform on Day 6 of rats. (j) Crossing the platform times. (k) Latency for rats to find the hidden platform. *: Comparison with age 6 and 18; #: comparison with age 12 and 18; δ: comparison with age 6 and 12; blue symbols indicate differences between males; red symbols indicate differences between females; &: gender difference of age 12. *, #, δ, &: *p* < 0.05; **, ##, δδ, &&: *p* < 0.01; ***, δδδ: *p* < 0.001; ****: *p* < 0.0001. *n* = 7–17 rats per group.

Three‐month‐old Sprague Dawley (SD) rats were obtained from Laboratory Animal Center, Guangdong University of Traditional Chinese Medicine and allowed to acclimate to the facility for at least 3 months prior to testing. All rats were housed (five rats per cage with corn‐cob bedding) in conventional laboratory rodent cages (ZS Dichuang Co., Beijing, China, dimensions 30.5 (W) * 22.5 (H) * 40.5 (L) cm) under standard conditions at 23°C–25°C and 40%–50% relative humidity with a 12 h light/dark cycle with water and food provided ad libitum. All animal procedures were approved by the Ethics Committee of Animal Research of South China Normal University (No. SCXK (Guangdong) 2016‐0041) and were carried out in accordance with the principles outlined in the NIH Guide for the Care and Use of Laboratory Animals (NIH Publications No. 8023, revised 1978). There were six groups in this study: 6‐month‐old female rats (F6), 6‐month‐old male rats (M6), 12‐month‐old female rats (F12), 12‐month‐old male rats (M12), 18‐month‐old female rats (F18), and 18‐month‐old male rats (M18). A timeline diagram of behavioral tests for the study is provided in Figure 1a. The rats were subjected to cognitive tests in a consistent order, including spontaneous alternation behavior, the light–dark discrimination test, and the Morris water maze, which took place with at least 24 h between them. After the behavioral tests, some rats (complete all behavioral tests) were used for Golgi–Cox staining, and some were used for other studies. All behavioral tests were performed by the same male experimenter.

### 2.2 Spontaneous Alternation Behavior (SAB)

Spontaneous alternation in the Y‐maze was used to assess short‐term spatial working memory. Before the experiment, rats were put into the room, which should be dark, quiet, and odorless. After the rat had adapted to the experimental environment for 30 min, we put the rat into an arm and took it out in 10 min. The sequence of the rats entering each arm in the maze was observed and recorded every minute. After the experiment, we took out the rat, cleaned up the feces and urine in the maze, sprayed into the maze with 75% alcohol, and dried them. In this experiment, two indexes are generally analyzed: the number of arms entries and the percentage of relative alternation. The arm entered by the rat differs from the two previously entered arms, which is defined as an alternation. The percentage of relative alternation was calculated from the ratio of the number of alternations divided by the number of entries.

### 2.3 Light–Dark Discrimination Test

Light–dark discrimination test is a training process in which the experimental animals are trained ten times a day for seven consecutive days, with a 24‐h interval between each training, which reflects the long‐term memory ability of animals. Before the experiment began, the rats were put into a dark and quiet room for 30 min to adapt. The experimental principle is to take advantage of rodents' nature of preferring darkness and avoiding light. When any of the three arms in the Y‐maze lights up, the darkened arm is electrically charged to see whether the rat would enter the bright arm. If the rat chooses the darkened arm, it received briefly electric footshocks (30 V AC, 0.45 mA; continued 1 s and started at 5 s after lamp‐bright) until it entered the bright arm. The commonly used indexes in this experiment are latency and accuracy. The latency is when the rat moves from the dark arm to the light arm after the light is turned on. The correct rate is the number of entering the bright arm/total number of entering the arm.

### 2.4 Morris Water Maze (MWM)

The Morris water maze experiment was used to test long‐term spatial memory. The device used in the experiment was a cylindrical tank with a diameter of 150 cm and a height of 50 cm, filled with water temperature kept at 20°C–22°C. There were four different shapes of graphic marks on the upper end of the water tank, and the water was evenly divided into four quadrants. A video system above the water is connected to a computer for real‐time analysis. Before the experiment began, the rats were put into a quiet room for 30 min to adapt. The experiment is divided into the train phase (5 days) and the test phase (1 day). Each rat was trained 4 times each day at intervals of 20 min. In the training phase, the rats were placed in the water to swim within 100 s. The latency for rats to find the hidden platform was recorded (rats that could not find the hidden platform within 100 s were excluded). In the test phase, the platform was removed and the rats were put into the water to swim within 60 s. The number of rats to cross the platform position and the ratio of the distance near the platform were recorded.

### 2.5 Golgi–Cox Staining

Golgi–Cox staining was performed as previously described [19]. After behavioral tests, the rats were decapitated, and their brains were removed and placed in the Golgi staining solution for 7 days. Then, the brains were transferred to the protective solution (5 mL protective solution), and the new solution was replaced once a day for seven consecutive days. The brains were immersed in paraffin and embedded. After the paraffin was solidified at room temperature, the brains were stored at −20°C overnight. On the second day, the slices were sectioned with a thickness of 150 μm and placed on 3% gelatin‐embedded slides to prevent falling off during the subsequent processing. The slides were washed with double distilled water for 1 min, immersed in 14% ammonia water for 30 min, washed with double distilled water three times for 1 min each time, immersed in 5% sodium thiosulfate for 10 min, and washed with double distilled water for two times for 1 min each time. They were engaged in 50%, 75%, and 95% ethanol for 4 min, then two times in absolute ethanol for 4 min each, transferred to xylene for 15 min, and sealed with neutral resin. Dendrites and spines from five neurons in the hippocampus's CA1, CA3, and DG areas of every rat were observed and photographed under a microscope (Leica, DM6). Sholl analysis plugin of ImageJ software was employed to assess the length and complexity of the dendrites. The dendritic spine density is expressed as the number of spines/dendritic segment lengths per 10 μm.

### 2.6 16S rRNA Gene Microbiome Analysis

Feces from each group of 6 rats were collected and transferred to a −80°C refrigerator. DNA extraction and purification were conducted with a MagaBio Soil/Feces Genomic DNA Purification Kit (Bioer, Hangzhou, China) according to the manufacturer's instructions. The 16S rRNA gene was amplified by PCR with the universal primers 515F (5‐GTGCCAGCMGCCGCGGTAA‐3) and 806R (5‐GGACTACHVGGGTWTCTAAT‐3) using a TaKaRa Premix Taq Version 2.0 (TaKaRa Biotechnology Co., Dalian, China) As instructed, the purified PCR products were sequenced using the Biomarker‐Technologies Company, China (PacBio) platform. SMRT‐Link (version 8.0) was used to obtain Circular Consensus Sequencing (CCS) sequences, and then, lima (v1.7.0) software was used to obtain high‐quality CCS sequences. UPARSE was used to classify high‐quality sequences into operational taxonomy units (OTUs) at a cutoff of 97% identity. Alpha diversity indices (Chao 1 and Shannon) were calculated for each sample, and beta diversity was analyzed using a principal coordinate analysis (PCoA) based on the uniface distance matrix to show differences in microbial community structure between populations. Linear discriminant analysis (LDA) was performed to identify biomarkers of microbiomes using Linear discriminant analysis Effect Size (LEfSe) software. The platform BMKCloud (www.biocloud.net) was used for alpha diversity, beta diversity, and metastatic analysis. The Illumina sequences reported here have been deposited in the NCBI Sequence Read Archive4 under accession number PRJNA54337.

### 2.7 Statistical Analysis

All data were expressed as mean ± standard error (mean ± SEM). Results with a normal distribution (*p* > 0.05 in Kolmogorov–Smirnov tests) were analyzed using Student two‐tailed *t*‐tests to compare two groups or one‐way or two‐way ANOVA followed by a Tukey's post hoc analysis for multiple comparisons using GraphPad Prism. Data that did not conform to a normal distribution were analyzed by Mann–Whitney test (nonparametric test). For all comparisons, *p* < 0.05 was set as statistically significant.

## 3 Results

### 3.1 Effects of Age and Gender on Cognitive in Rats

We assessed the cognitive ability of rats using the Y‐maze SAB test, the light/dark discrimination test, and the MWM test (Figure 1). In the SAB test, there was no difference in the percentage of relative alternation rate among all groups (Figure 1c). For the number of arms entries, Tukey's post hoc multiple comparison of two‐way ANOVA revealed that 18‐month‐old rats (F18 and M18) showed a significant decrease compared with 6‐month‐old rats (F6 and M6) by gender (Figure 1d). Besides, M12 exhibited a significant increase compared with M18 and F12. In the light–dark discrimination test, the correct ratio of F18 was decreased significantly on the first day (Figure 1f). Compared with M6, M12 exhibited a significant decrease on Days 2, 5, 6, and 7, and likewise, M18 exhibited a significant decrease on Days 6 and 7 (Figure 1f). The latency of F18 was longer dramatically than F6 and F12 from Days 2 to 7, and the longer latency was seen in M18 than in M6 on Days 2–7 (Figure 1g). In the MWM test, the ratio of the distance near the platform of 18‐month‐old rats (F18 and M18) significantly decreased compared with 6‐month‐old rats (F6 and M6) by gender (Figure 1i). For the crossing times, just M18 showed a significant decrease (Figure 1j). For the latency, F12 exhibited a significant increase on the first day compared with M12. These findings suggested that the cognitive ability of rats gradually declined with aging, and the cognitive ability of females was worse than the males for 12‐month‐old rats.

### 3.2 Effects of Age and Sex on Dendrite Structure and Dendrite Spine Density in Hippocampal CA1 Region of Rats

**FIGURE 2:**
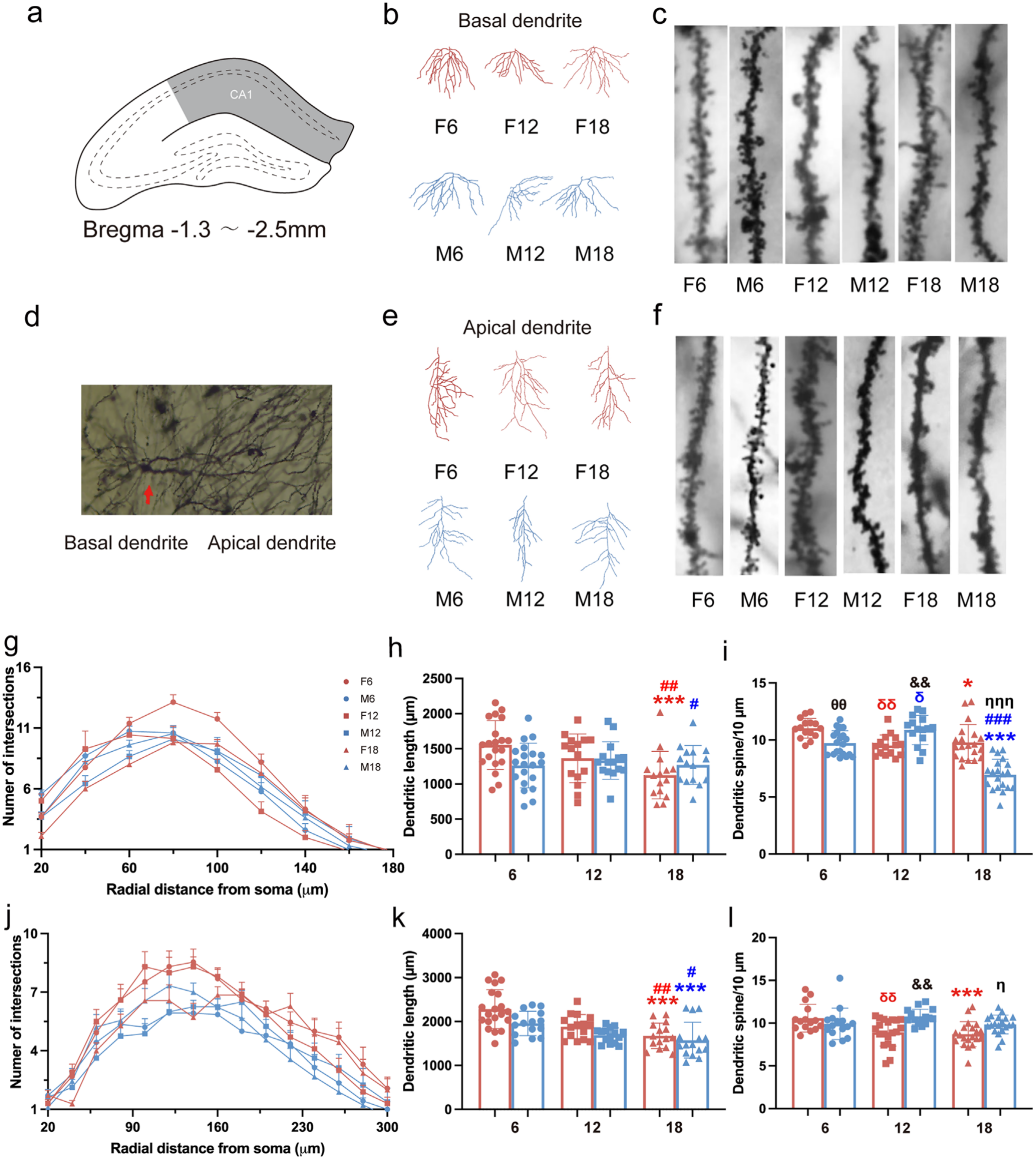
Effect of age and sex on the morphology of neurons in hippocampal CA1 region in rats. (a) Schematic diagram of the location of the CA1 region. (b) The basal dendrites in the CA1 region of the hippocampus. (c) The spine density of the basal dendrites. (d) Neurons in the CA1 region of the hippocampus. (e) The apical dendrites in the CA1 region of the hippocampus. (f) The spine density of the apical dendrites. (g) Number of the intersections of basal neurons at a distance from the soma in rats at different months of age. (h) The total length of basal neuron dendrites. (i) The spine density of basal dendrites. (j) Number of the intersections of apical neurons at a distance from soma in rats at different months of age. (k) The total length of basal neuron dendrites. (l) The spine density of dendrites. *: Comparison with age 6 and 18; #: comparison with age 12 and 18; δ: comparison with age 6 and 12; blue symbols indicate differences between males; red symbols indicate differences between females; θ: gender difference of age 6; &: sex difference of age12; η: sex difference of age 18. *, #, δ, &, θ, η: *p* < 0.05; **, ##, δδ, &&, θθ, ηη: *p* < 0.01; ***, ###, δδδ, &&&, θθθ, ηηη: *p* < 0.001. Every dot presents one neuron (*n* = 15 or 20) from rats (*n* = 3 or 4).

The CA1 region (Figure 2a) is mainly responsible for short‐term memory [20]. We quantitatively analyzed the dendritic morphology and dendritic spine density of the hippocampal CA1 region of rats (Figure 2b–f) by Tukey's post hoc multiple comparisons of two‐way ANOVA. For basal dendrite, the number of intersections by Sholl analysis is shown in Figure 2g. The total dendritic length of F18 was significantly shorter than F6 and F12. (Figure 2h). For the spine density of basal dendrite, M18 was remarkably decreased compared to M6, M12, and F18; F6 was significantly increased compared to F12, F18, and M6; and M12 was significantly increased compared to F12 (Figure 2i). For apical dendrites, the number of intersections by Sholl analysis is shown in Figure 2j. The total length of F18 was shorter than F6 and F12, and the total length of M18 was shorter than M6 and M12 (Figure 2k). For the spine density of apical dendrite, F6 was significantly increased compared with F12 and F18; M12 and M18 were significantly increased compared to F12 and F18, separately (Figure 2l). These results suggested that the neuronal dendritic structures of CA1 region of the hippocampus were damaged in old aged rats; the spine loss of females occurred earlier than that of males.

### 3.3 Effects of Age and Sex on Dendrite Structure and Dendrite Spine Density in Hippocampal CA3 Region of Rats

**FIGURE 3:**
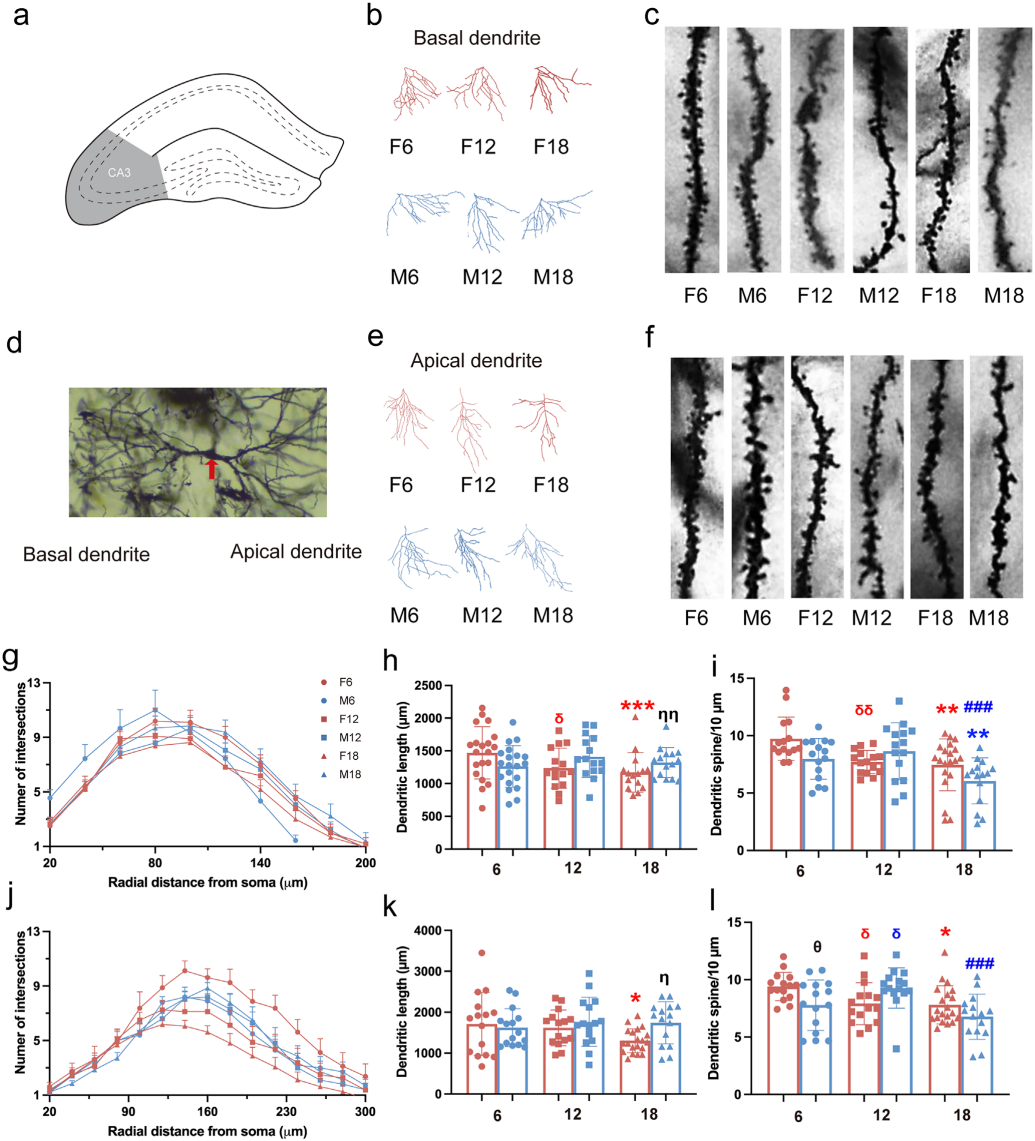
Effect of age and sex on the morphology of neurons in hippocampal CA3 region in rats. (a) Schematic diagram of the location of the CA3 region. (b) The basal dendrites in the CA3 region of the hippocampus. (c) The spine density of the basal dendrites. (d) Neurons in the CA3 region of the hippocampus. (e) The apical dendrites in the CA3 region of the hippocampus. (f) The spine density of the apical dendrites. (g) Number of the intersections of basal neurons at a distance from the soma in rats at different months of age. (h) The total length of basal neuron dendrites. (i) The spine density of basal dendrites. (j) Number of the intersections of apical neurons at a distance from soma in rats at different months of age. (k) The total length of basal neuron dendrites. (l) The spine density of dendrites. *: comparison with age 6 and 18; #: comparison with age 12 and 18; δ: comparison with age 6 and 12; blue symbols indicate differences between males; red symbols indicate differences between females; θ: sex difference of age 6; &: sex difference of age 12; η: sex difference of age 18. *, #, δ, &, θ, η: *p* < 0.05; **, ##, δδ, &&, ηη: *p* < 0.01; ***, ###, δδδ, &&&: *p* < 0.001. Every dot presents one neuron (*n* = 15 or 20) from rats (*n* = 3 or 4).

The CA3 region (Figure 3a) is the most critical brain region in the formation of associative memory [21]. The dendritic morphology and dendritic spine density of the hippocampal CA3 region of rats (Figure 3b–f) were quantitatively analyzed by Tukey's post hoc multiple comparison of two‐way ANOVA. For basal dendrite, the number of intersections by Sholl analysis is shown in Figure 3g. The total dendritic length of F6 was significantly longer than F12 and F18, and the total dendritic length of M18 was significantly longer than F18 (Figure 2h). For the spine density of basal dendrite, F12 and F18 were remarkably decreased compared to F6, and M18 was significantly decreased compared with M6 and M12 (Figure 3i). For apical dendrites, the number of intersections by Sholl analysis is shown in Figure 3j. The total dendritic length of F6 was significantly longer than F18, and the total dendritic length of M18 was significantly longer than F18 (Figure 3k). For the spine density of apical dendrite, F12 and F18 were remarkably decreased compared to F6; M18 was also remarkably decreased M12; and M6 was decreased compared with F6 significantly (Figure 3l). These results suggested that the neuronal dendrites of CA3 region of the hippocampus were damaged in old‐aged rats. The spine loss of female was more than the male for twelve‐month‐old rats.

### 3.4 Effects of Age and Sex on Dendrite Structure and Dendrite Spine Density in Hippocampal DG Region of Rats

**FIGURE 4:**
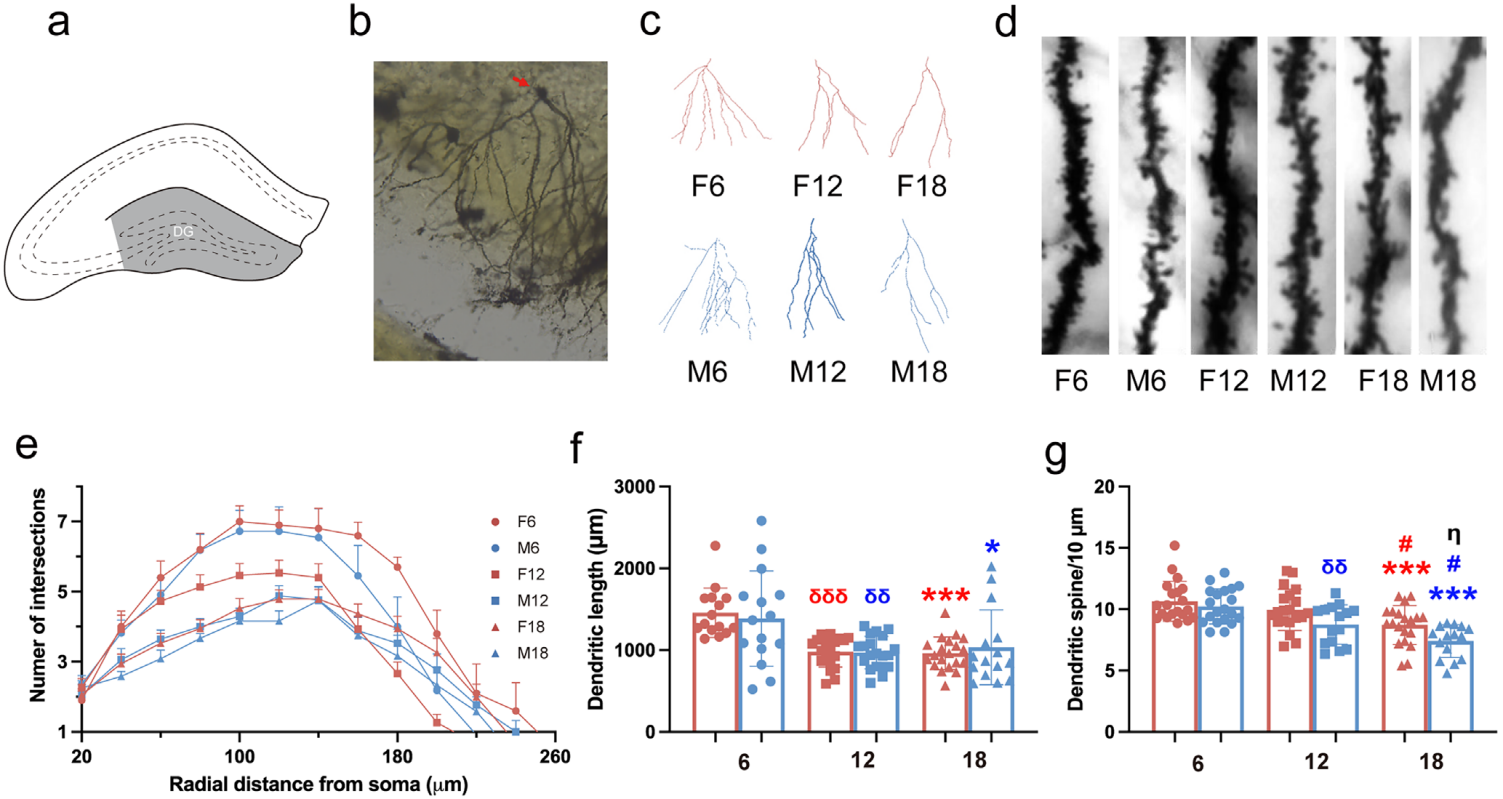
Effect of age and sex on the morphology of neurons in hippocampal DG region in rats. (a) Schematic diagram of the location of the DG region. (b) Neurons in the DG region of the hippocampus. (c) Neuron structure in the DG region. (d) The spine density of dendrites. (e) Number of the intersections of neurons at a distance from the cell body in female rats at different months of age. (f) The total dendritic length in the DG region. (g) The spine density of dendrites. *: comparison with age 6 and 18; #: comparison with age 12 and 18; δ: comparison with age 6 and 12; &: sex difference of age 12; blue symbols indicate differences between males; red symbols indicate differences between females; *, #, δ, &: *p* < 0.05; **, ##, δδ: *p* < 0.01; ***, ###, δδδ: *p* < 0.001. Every dot presents one neuron (*n* = 15 or 20) from rats (*n* = 3 or 4).

The DG region (Figure 4a) serves critical functions in cognition [22]. We quantitatively analyzed the dendritic morphology and dendritic spine density of the hippocampal DG region of rats (Figure 4b–d) by Tukey's post hoc multiple comparison of two‐way ANOVA. For the dendrite, the number of intersections by Sholl analysis is shown in Figure 4e. For the total dendritic length, F6 was remarkably longer than F12 and F18, and M6 was significantly longer than M12 and M18 (Figure 4f). For the spine density of dendrite, M18 and M12 were remarkably decreased compared to M6; F18 was remarkably decreased compared to F12 and F6; and M18 was also remarkably decreased F18 (Figure 4g). These results suggested that the neuronal dendritic structures of DG regions of the hippocampus were damaged in rats with aging.

### 3.5 Effects of Age and Sex on Gut Microbiota in Rats

**FIGURE 5:**
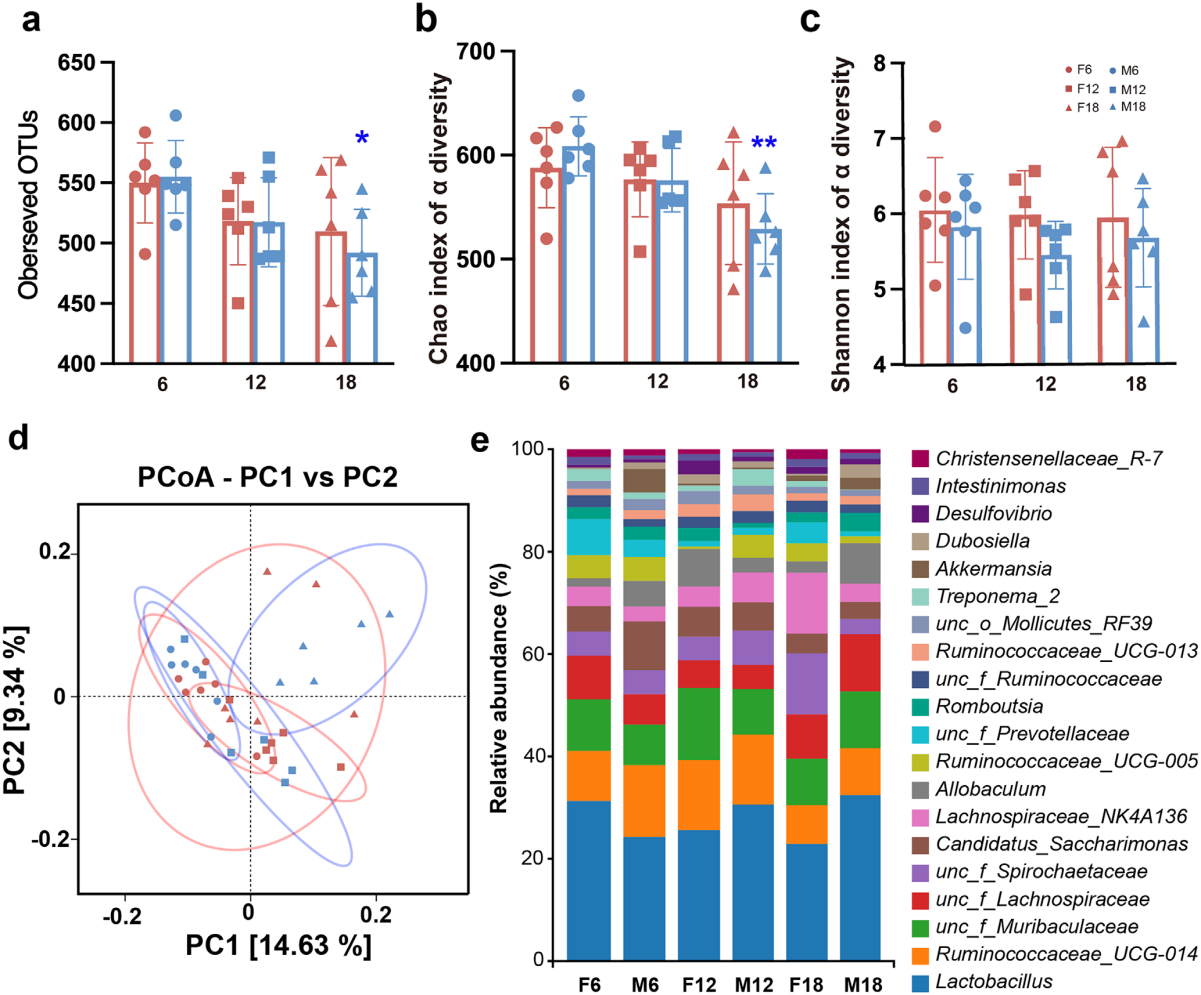
Effects of age and sex on diversity and composition of gut microbiota in rats. (a) Observed OTUs. (b) Chao index of α‐diversity. (c) Shannon index of α‐diversity. (d) Principal component analysis (PCoA) of weighted UniFrac distance in OTUs level. (e) Microbiome composition at the level of genera. *: comparison with M6 and M18. **: *p* < 0.01; *n* = 6.

A total of 36 fecal samples were analyzed by 16S rRNA gene sequencing to investigate the gut microbiota. To investigate how gender and age affect the gut microbiota richness and diversity in each fecal sample, we analyzed the α‐diversity (Figure 5a–c). The Chao1 index represents the number of taxa in the sample predicted by inferring the number of rare organisms that may have been missed due to the small sample size. The Shannon index is used to measure microbiota diversity and is influenced by species richness and evenness. Interestingly, the observed OTUs and Chao1 index of M18 were smaller than M6. Principal coordinate analysis (PCoA) was applied to illustrate the distribution of the microbial community in the samples at the OTU level (Figure 5d). The first principal coordinate (PC1, *x*‐axis) explains 14.63% of the variation in the data, while the second principal coordinate (PC2, *y*‐axis) decreases the total explained variation to 9.34%; M18 had no overlap in the microbiota with M12 and M6, along with the analysis of similarity in the distribution (ANOSIM, M18:M6, *R* = 0.630, *p* = 0.008; M18:M12, *R* = 0.519, *p* = 0.008), which indicates M18 is different with M12 and M6 and presents dissimilarity in RNA expression among them. We then analyzed the composition of microbiota communities at the genus level, and genera with the top 20 abundances are shown in Figure 5e.

**FIGURE 6:**
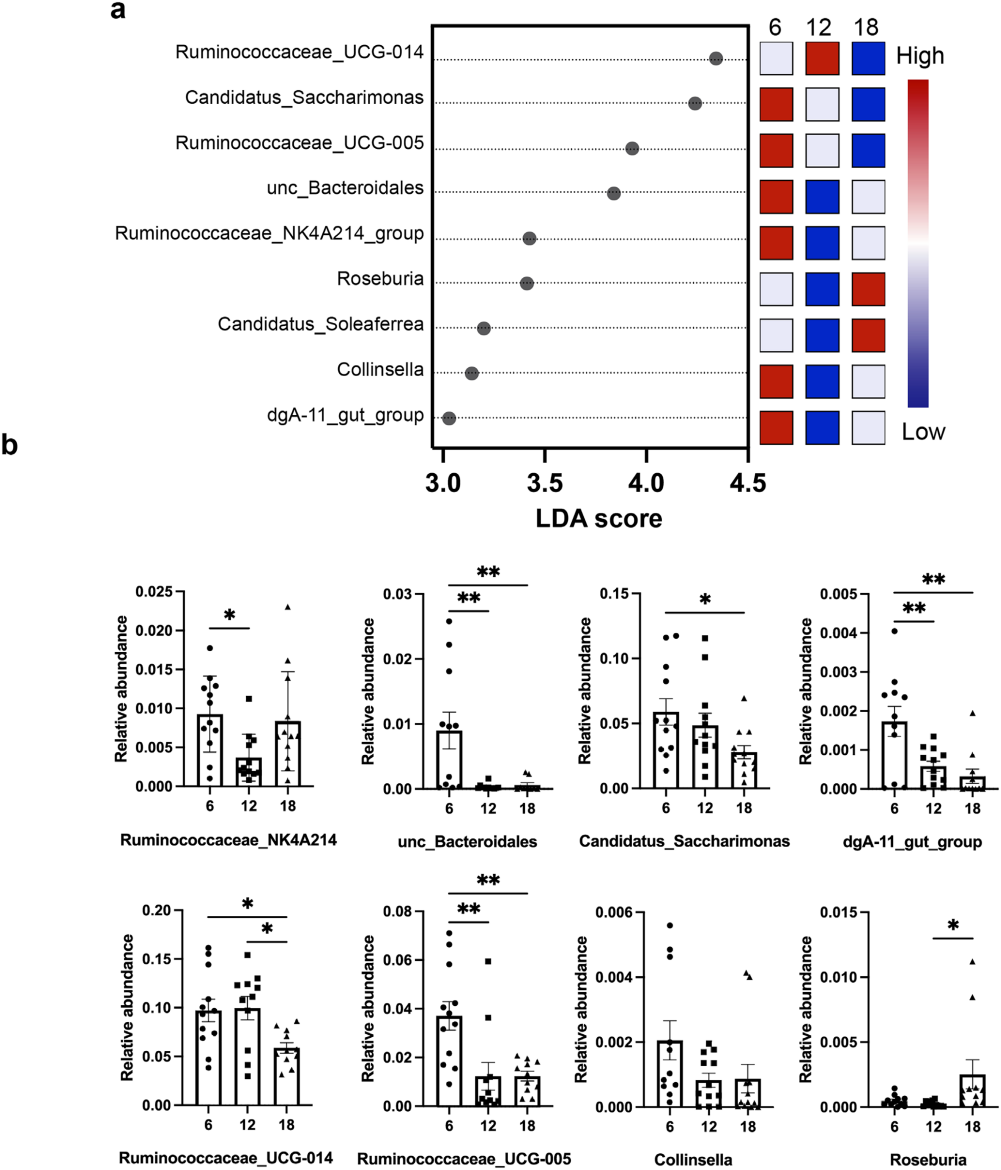
Gut microbiota associated with aging in genus level. The left histogram shows the Linear discriminant analysis (LDA) Effect Size (LEfSe) scores computed for features (in the genus level) differentially abundant between the different age groups. (a) The right heatmap shows the relative abundance (log10 transformation) of genera. (b) Top general alterations in aging. “unc”: uncultured bacterium. *: *p* < 0.05; **: *p* < 0.01; *n* = 12.

**FIGURE 7:**
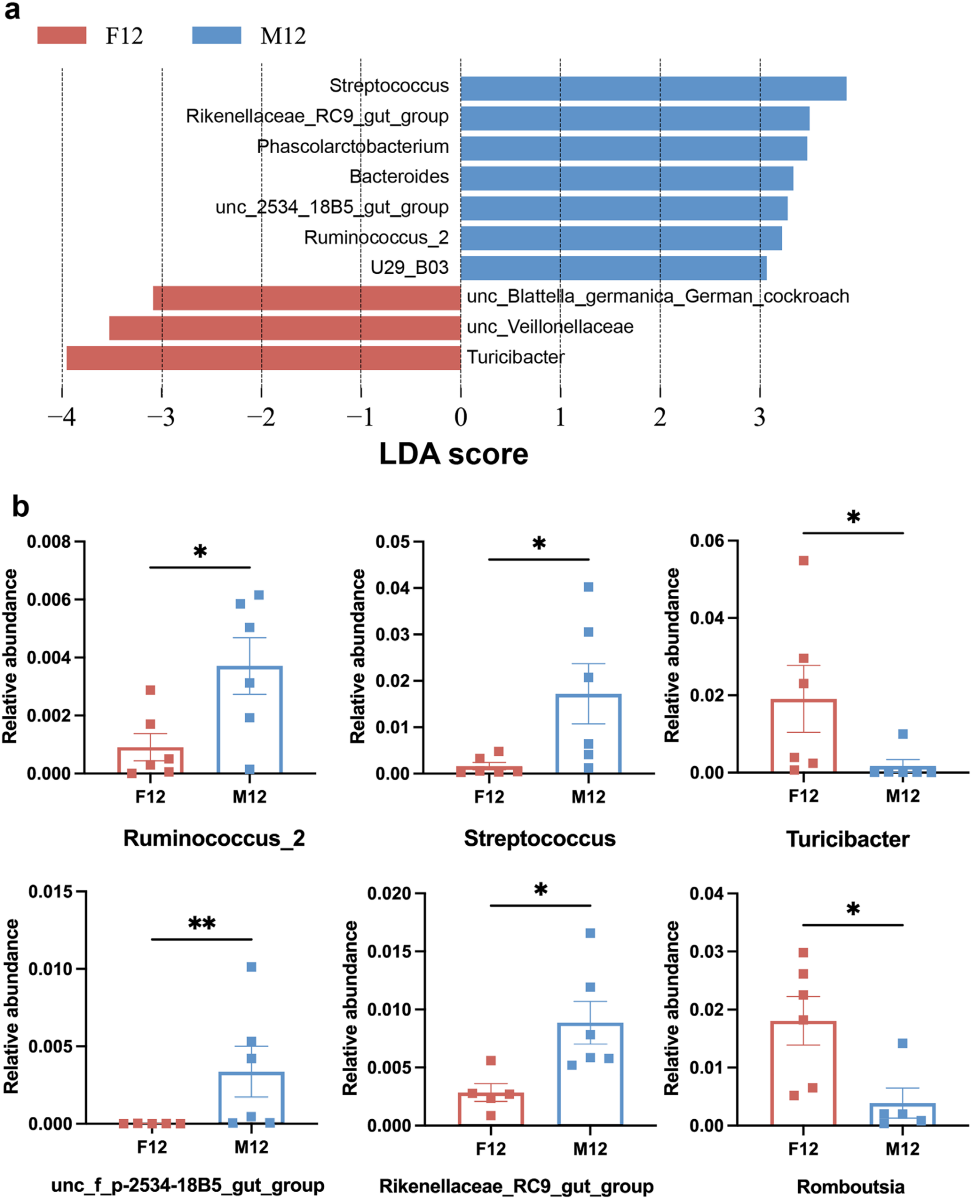
Gut microbiota associated with the gender of middle‐aged rats in genus level. (a) Cladogram using the LEfSe method indicating the general distribution of gut microbiota associated with M12 (blue) and F12 (red). (b) Top general alterations with the gender of middle age rats. “unc”: uncultured bacterium. *p < 0.05; **p < 0.01; *n* = 6.

To identify specific bacteria that differ in age at the genus level, we applied LDA to identify 9 significant genera as shown in Figure 6a. They were quantitatively analyzed by one‐way ANOVA followed by Tukey's post hoc analysis. There were age‐dependent decreases in *unc‐Bacteroidales, Candidatus_Saccharimonas, dgA‐11_gut_group, Ruminococcaceae* UCG‐014, and *Ruminococcaceae* UCG‐005 (Figure 6b). On the contrary, there was an age‐dependent increase in *Roseburia* (Figure 6b). To identify specific bacteria that differ in gender at the genus level, we applied LDA to identify seven significant genera in M12 and three significant genera in F12 as shown in Figure 7a. The gut microbiomes of the M12 were characterized by a preponderance of *Ruminococcu_2, Streptococcus*, *unc fp‐2534‐18B5_gut_group*, *Phascolarctobacterium, Bacteroides, U29 B03*, and *Rikenellaceae_RC9_gut_group in genus level*, whereas the microbiomes were characterized by a preponderance of *unc_Blattella_germanica_Germanjcockroach, unc Veillonellaceae*, and *Turicibacter* in F12. The relative abundance of *Ruminococcu_2, Streptococcus*, *unc fp‐2534‐18B5_gut_group*, and *Rikenellaceae_RC9_gut_group* was significantly increased in male than female rats (Figure 7b). The relative abundance of *Turicibacter* and *Romboutsia was* significantly decreased in male than female rats (Figure 7b). These results indicated that the microbial diversity of gut microbiota was significantly decreased in old‐aged male rats; some specific bacteria of microbiota communities were changed with aging; and some specific bacteria differed in gender for middle‐aged rats.

## 4 Discussion

The present study found that the cognitive ability of old‐age rats was decreased than adult and middle‐aged rats; males have better cognitive ability than females for middle‐aged rats. The neuronal dendritic structures of the hippocampus were damaged to different degrees during aging, and spine loss occurred in females rather than males in middle‐aged rats. In addition, the microbial diversity of gut microbiota was significantly decreased in old‐aged male rats; some specific bacteria of microbiota communities were changed with aging; and some specific bacteria differed in gender for middle‐aged rats.

A series of behavioral tests were conducted to study the cognition of naturally aged rats of different genders. SAB test has been widely accepted as a quick and simple test of memory because it avoids the need for extensive training [23]. As expected, old‐aged rats showed decreased short‐term memory ability. In particular, female rats exhibited a significant decrease in short‐term memory ability compared with male rats in middle age. However, recent studies showed that proestrus mice performed well in SAB, but this phenomenon was not seen in mice with low estrogen [24, 25]. Likewise, female mice were significantly increased as compared with the male mice at 6 weeks old [26]. These apparently conflicting findings might be due to different ages of rodents and disturbances in estrogen levels. Light–dark discrimination test in the Y‐maze is a more difficult and complicated learning task for rats or mice than the SAB test and usually serves as a long‐term memory test [19]. The results showed that the cognitive ability of rats gradually declined with aging, which is consistent with previous findings that aged wild‐type mice showed impaired spatial memory retention in the Y‐maze [27]. The MWM tests spatial memory in rodents [28]. The results showed that old‐aged rats showed impaired spatial memory, and there are gender differences in spatial memory in middle‐aged rats. It was revealed that aged mice display impaired spatial memory in the MWM [29], which is consistent with our findings. In particular, female rats exhibited a significant decrease in spatial memory in the MWM compared with male rats in middle age. Similarly, a previous study showed that male rats had significantly shorter latency to find the hidden platform than female rats during training days [30]. In addition, gender divergences in spatial memory appear when task difficulty increases [31]. Overall, these results support that spatial memory becomes inaccurate with aging and gender is one of the important factors.

Cognitive is reflected not only in behavior, but also in changes in the structure and function of neurons. The structural plasticity of dendrites and spines of neurons is widely believed to underlie memory [32]. Previous studies have revealed that loss of dendritic spines has been demonstrated in rodents with cognitive impairment [5, 6]. Our results showed that neuronal dendritic structures of the hippocampus were damaged to different degrees in hippocampal CA1, CA3, and DG regions during natural aging. Similarly, it was showed that there were reductions in the density of dendritic spines in aged animals [33]. Besides, a significant decrease in dendritic spine density was observed in 9‐month‐old mice when compared with 3‐month‐old mice [34], which means that synaptic disorders occurred before cognitive impairment was discovered. There are similar findings that middle‐aged female mice did not show cognitive impairment in maze tests, but the total length of dendrites and the density of dendritic spines in the hippocampus decreased in our study. Furthermore, the present study demonstrated that there is a significant difference in spine loss between middle‐aged female and male rats; this difference disappeared during aging. Consistently, gender differences in dendritic branching and apical spine density of CA1 pyramidal cells were found in young adults rather than aged animals [35]. However, another study found more dendritic spines in the CA3 pyramidal neurons in females than in males [36]. These apparently conflicting findings might be due to the differences in subregion and age, which suggests that we should carefully choose the age and gender of animals in the study. In addition, our results showed that the dendritic complexity of neurons in the DG region declined particularly obviously as early as female and male middle‐aged rats. The hippocampal DG region is widely severed as a specific area where new neurons are continuously regenerated from neural stem cells in the adult brain, which is closely related to learning and memory [37, 38]. As a result, a more obvious response to aging was found in the DG region, which outweighed the gender difference.

Gut microbiota is symbiotic with humans and affects our health, and its role has been paid more and more attention. Human studies have shown that the diversity of the gut microbiome altered, and the abundance of *Firmicutes* decreased and that of *Bacteroides* increased with age [39, 40]. However, the age‐related variations in gut microbiota have not been consistent. For example, studies showed the microbial composition and diversity of the gut ecosystem of young adults and 70‐year‐old people were highly similar [13, 41]. In contrast, Odamaki et al. reported that they observed an increase in gut microbiota diversity with aging until the centenarian stage [9]. Rodents are an excellent model for studying gut microbiome, due to the lack of influence of dietary preferences and nationality. Our results indicated that the microbial α‐diversity of gut microbiota was significantly decreased in old‐aged male rats, which was consistent with that the number of gut microbiota species gradually decreased with aging [11]. These results highlight the biology of aging and the intricate interactions between the host and microbiota during aging. Recent animal studies have indicated that the gut microbiota affects cognition [11, 42].

Our understanding of the relationship between the gut microbiota and cognitive function during aging is limited because the biology of the gut flora during aging is not considered. In order to explore the effect of gut microbiota on cognitive dysfunction in aging, we explored changes in gut microbiota at 6, 12, and 18 months of age to identify gut microbiota on the longitudinal axis of time that were consistent with cognitive changes in rats. At the genus level, the relative abundance of *unc‐Bacteroidales, Candidatus_Saccharimonas, dgA‐11_gut_group, Ruminococcaceae UCG‐014, and Ruminococcaceae UCG‐005* gradually declined with aging, and their decrease might lead to cognitive impairment. The bacterial genus *Candidatus_Saccharimonas*, which belongs to the phylum *Patescibacteria*, inhibited the production of anti‐inflammatory cytokines (IL‐2, IL‐4, IL‐10, and IFN‐γ) [43, 44], which led gut microbiota of older age represents a pro‐inflammatory phenotype. In the process, permeable BBB and neuroinflammation led to neural injury and ultimately a reduction in cognitive function [11]. The bacterial genus *Ruminococcaceae UCG‐014, and Ruminococcaceae UCG‐005*, which belongs to the *Ruminococcaceae* family, attenuated the neuronal loss and cognitive deficits of aged mice [45]. However, there was an increase in *Roseburia* in old‐aged rats. The bacterial genus *Roseburia*, which is butyrate‐producing bacteria in the host colon from the *Lachnospiraceae* family, has also been highlighted in protection of the nervous system from diseases [46]. While some microbiota related to human studies have shown partially contradictory results, it is hoped that gut microbiota in age longitudinal studies in the future will make it possible to use them as biomarkers for gut microbiota dysbiosis and cognitive impairment in the future.

Despite the importance of the gut microbiota to human health has been noted for decades, few studies have addressed the impact of gender on the gut microbiota in human, particularly gender differences in the aging process. In general, the composition of the gut microbiota appears to vary by gender, and the diversity appears to be bigger in females [47]. However, in some studies, there was no significant difference in the α‐diversity between males and females [48, 49]. The study also did not find gender differences in microbiota diversity of the same age groups. In order to further identify microbial differences between genders, we performed LDA coupled with effect size measurements (LEfSe). At the genus level, the relative abundance of *Streptococcus*, *Ruminococcus_2, unc fp‐2534‐18B5_gut_group*, *Rikenellaceae_RC9_gut_group, Turicibacter*, and *Romboutsia* was significantly different between male and female rats at middle age. Consistently, the study found sex differences in cognitive ability in 12‐month‐old rats, which may be attributed to the above differences in the abundance of gut microbiota. *Streptococcus* is a significant cause of sepsis, meningitis, and death in infants under 3 months of age worldwide [50], which could produce serotonin. *Ruminococcus* and *Rikenellaceae RC9 gut group* plays an important role in the digestion of crude fiber [51]. *Ruminococcus* were positively correlated with the level of serum testosterone [49]. *Turicibacter* and *Romboutsia* are positively related to the immune response by increasing inflammatory cytokines [52]. Increases in beneficial flora and decreases in pro‐inflammatory flora led 12‐month‐old male rats to come with healthier gut microbiota, which may explain their higher cognitive levels than female rats. Understanding how gender differences affect the gut microbiota could ultimately lead to the identification of new factors affecting disease susceptibility and improved diagnostic and clinical strategies. In the future, we can use estrogen intervention, gut microbiota transplantation, or antibiotics to regulate gut microbiota to observe whether the gut microbiota affected the gender difference in cognition of rats with aging.

In conclusion, our results suggested that cognitive impairment in aged rats might result from dendritic damage in the hippocampus and gut microbiota dysbiosis, which provide direct evidence that gender differences in dendritic damage and gut microbiota dysbiosis contribute to cognitive impairment in naturally aged rats.

## Author Contributions

K.L. designed and performed Golgi staining and behavior experiments. Y.‐Y.H. and J.‐Y.H. analyzed the data. Y.‐Y.H. and J.‐C.W. wrote the paper. D.‐X.L. provided suggestions for the manuscript revision. C.‐H.L. supervised the study, designed experiments, and critically revised the manuscript.

## Conflicts of Interest

The authors declare no conflicts of interest.

## Data Availability

The data that supports the findings of this study are available in the Supporting Information of this article.
